# Molecular Imaging-Guided Interventional Hyperthermia in Treatment of Breast Cancer

**DOI:** 10.1155/2015/505269

**Published:** 2015-09-30

**Authors:** Yurong Zhou, Jihong Sun, Xiaoming Yang

**Affiliations:** ^1^Department of Radiology, Sir Run Run Shaw Hospital, Zhejiang University School of Medicine, 3 East Qingchun Road, Hangzhou, Zhejiang 310016, China; ^2^Image-Guided Bio-Molecular Interventions Research, Department of Radiology, University of Washington School of Medicine, 815 Mercer Street, Room S470, Seattle, WA 98109, USA

## Abstract

Breast cancer is the most frequent malignancy in women worldwide. Although it is commonly treated via chemotherapy, responses vary among its subtypes, some of which are relatively insensitive to chemotherapeutic drugs. Recent studies have shown that hyperthermia can enhance the effects of chemotherapy in patients with refractory breast cancer or without surgical indications. Recent advances in molecular imaging may not only improve early diagnosis but may also facilitate the development and response assessment of targeted therapies. Combining advanced techniques such as molecular imaging and hyperthermia-integrated chemotherapy should open new avenues for effective management of breast cancer.

## 1. Introduction

Breast cancer is the most common malignancy in women worldwide. Although chemotherapy has improved the survival of breast cancer patients, some breast cancer subtypes are relatively insensitive to chemotherapy drugs. Breast cancers are highly heterogeneous, comprising different histologic tissues as well as gene expression and mutation patterns [1]. In patients with refractory breast cancer or without surgical indication, hyperthermia (mild heat at approximately 42°C) may augment chemotherapy. Although thermal ablation (temperatures above 60°C) can directly destroy cancer tissue [2, 3], it has shown limited success in tumors located adjacent to normal structures, such as blood vessels, which are prone to thermal injury [4]. Incomplete ablation also frequently occurs at treated tumor margins owing to heat dissipation by neighboring blood flow or incomplete coverage of large, irregularly shaped tumors by the ablation field. Because of these drawbacks, thermal ablation often results in tumor recurrence.

Radiofrequency- (RF-) mediated, nonablative local hyperthermia with body-tolerable temperatures of 40–44°C can significantly enhance the antitumor effects of different drugs in a variety of malignancies [5–9]. It may do so by heating fractured tissue or increasing cellular metabolism, the permeability of cytoplasmic membranes, or the activity of heat shock proteins [10, 11]. All of these mechanisms facilitate the entrance of therapeutics into the targeted tumor cells for effective destruction of tumor tissue. Motivated by the success of RF-mediated, nonablative hyperthermia, an innovative interventional oncologic technique termed “percutaneous intratumoral RF hyperthermia-enhanced chemotherapy of cancers” has been introduced [5–7]. This technique combines hyperthermia and chemotherapy and has increased survival in several randomized clinical trials [12–15]. For example, the 10-year disease-free survival rate was 53–72% in bladder cancer patients receiving a combination of hyperthermia and chemotherapy compared with 15% in those receiving chemotherapy alone [16, 17].

Molecular imaging is one of the frontiers in modern medical imaging [18]. It can detect biological events occurring at the cellular and molecular levels in vivo and has demonstrated great potential in the early diagnosis and effective treatment of a number of life-threatening diseases including breast cancer. Molecular imaging can be potentially used to screen patients, stage tumors, guide therapy, and assess responses to therapy [19]. Furthermore, recent development of a molecular imaging-guided, RF hyperthermia-enhanced interventional oncology technique has facilitated the efficient management of breast cancer by combining molecular imaging and hyperthermia technology-integrated chemotherapy [20]. In this paper, we review the current status of molecular imaging-guided hyperthermia in breast cancer treatment.

## 2. Molecular Subtypes and Targeted Therapies in Breast Cancer

To date, breast cancer has four main subtypes: luminal A, luminal B, basal-like, and human epidermal growth factor 2- (HER2-) enriched [21]. This classification is based on information gathered from different molecular assays including DNA methylation assays, exome sequencing, microRNA sequencing, and DNA copy number, mRNA, and reverse-phase protein arrays (Table 1) [21, 22]. The different subtypes have different molecular characteristics and different sensitivities to chemotherapy. The luminal A subtype has the best prognosis after chemotherapy, followed by the luminal B subtype, while the basal-like and HER2-enriched subtypes have the worst prognosis [23]. The luminal A and B subtypes are amenable to hormone therapy, and the HER2-enriched subtype is a potential candidate for trastuzumab therapy [24].

The primary goal of targeted therapy—killing tumor cells while sparing surrounding healthy cells—is the basis of personalized medicine. Personalized medicine in breast cancer has two primary components: targeted diagnosis and targeted treatment. The goal of targeted diagnosis is to accurately differentiate the subtypes of breast cancer according to the patient's molecular profile to ensure that the most effective treatments are selected. The goal of targeted treatment is to produce therapeutic-carrying probes that specifically target molecules uniquely expressed or overexpressed in tumors, as identified via molecular profiling, and thereby effectively treat the targeted lesions without damaging surrounding healthy tissues. Manufacturing probes with imaging-visible materials, such as metals for X-ray imaging, nucleoids for nuclear imaging, and heavy metals for magnetic resonance (MR) imaging (MRI), also achieves targeted imaging [25, 26]. Even with the current technologies, the specific targeting of systemically administered imaging and therapeutic probes is still limited because the molecular targets overexpressed in lesions may also be expressed at certain levels in healthy tissues.

Interventional radiology, which uses images to guide minimally invasive interventions, can help deliver targeted probes directly to targeted lesions, thus avoiding systemic toxicity of the probes in vital normal organs [27]. It can also expand the capabilities of currently available molecular imaging techniques to (i) reach deep-seated targets, (ii) enable a close look at small targets, (iii) precisely guide delivery of nontargeted imaging tracers or therapeutics, and (iv) selectively enhance the effectiveness of targeted imaging and treatment [28, 29].

## 3. Molecular Imaging in Breast Cancer

Molecular imaging is one of the hottest topics in modern medical imaging. Molecular imaging modalities can assess biologic processes at the molecular and cellular level, which is useful for early detection of cancer and complements traditional anatomic imaging methods [30]. Molecular imaging can be potentially used for breast cancer screening, staging, and guiding and assessing responses to therapy [19]. Techniques for molecular imaging of breast cancers include MRI, optical imaging, radionuclide imaging with positron emission tomography (PET) or single photon emission computed tomography (SPECT), and contrast-enhanced ultrasound imaging [19].

**Figure 1:**
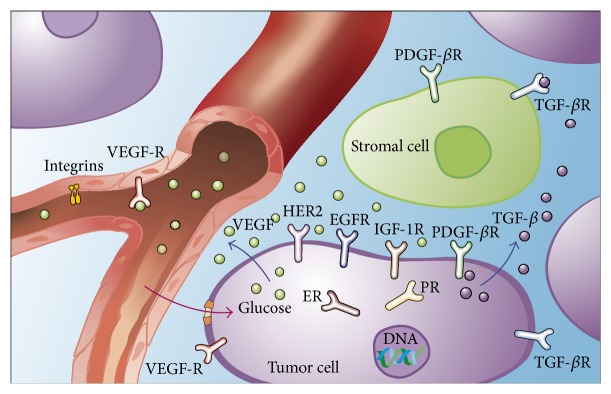
Schematic presentation of the (potential) targets for breast cancer molecular imaging. Reprinted, with permission, from [19].

For molecular imaging of breast cancer, proteins (e.g., receptors) overexpressed in breast tumors are starting points for the development of tumor-specific tracers (Figure 1) [19]. Hormone receptors are expressed in most breast tumor cells, and many receptors (e.g., HER2, epidermal growth factor receptor (EGFR), insulin-like growth factor-1 receptor, and platelet-derived growth factor *β* receptor) are present in the plasma membranes of tumor cells, which facilitates targeting because the probes do not need to enter cells. Receptor ligands, such as vascular endothelial growth factor (VEGF) and transforming growth factor *β*, are secreted by tumor cells into the tumor microenvironment, which allows regional molecular imaging of tumors. In addition, proteins (e.g., VEGF receptors, EGFRs, *α*_*v*_ *β*_3_-integrin, fibronectin, and endostatin) and conditions (e.g., hypoxia) involved in angiogenesis play important roles in breast tumor growth and thus are reasonable targets for molecular imaging of breast tumors [19]. Tumor cells have a higher metabolism and proliferation rate than do normal cells.

### 3.1. Molecular MRI

Conventional dynamic contrast-enhanced (DCE) or contrast-enhanced MRI of the breasts involves an intravenous injection of a low molecular weight T1-shortening paramagnetic contrast agent or dye. The rate of contrast agent uptake into breast lesions is nonlinear and differs between malignant and benign lesions. How a tumor responds to treatment is indicated by changes in the parameters of DCE-MRI. Diffusion-weighted- (DW-) MRI is based on the movement of water over distances of 0 to 30 *μ*m in 50 to 100 ms [31]. Differentiation between malignant and benign breast tumors via DW-MRI has been reported, with the mean apparent diffusion coefficient of malignant lesions being significantly lower than that of benign lesions or normal breast tissue [32, 33]. Breast tumors are less hypoxic than normal breast tissue [34]. Blood oxygen level-dependent or intrinsic susceptibility-weighted MRI relies on the paramagnetic property of deoxyhemoglobin [35].

**Figure 2:**
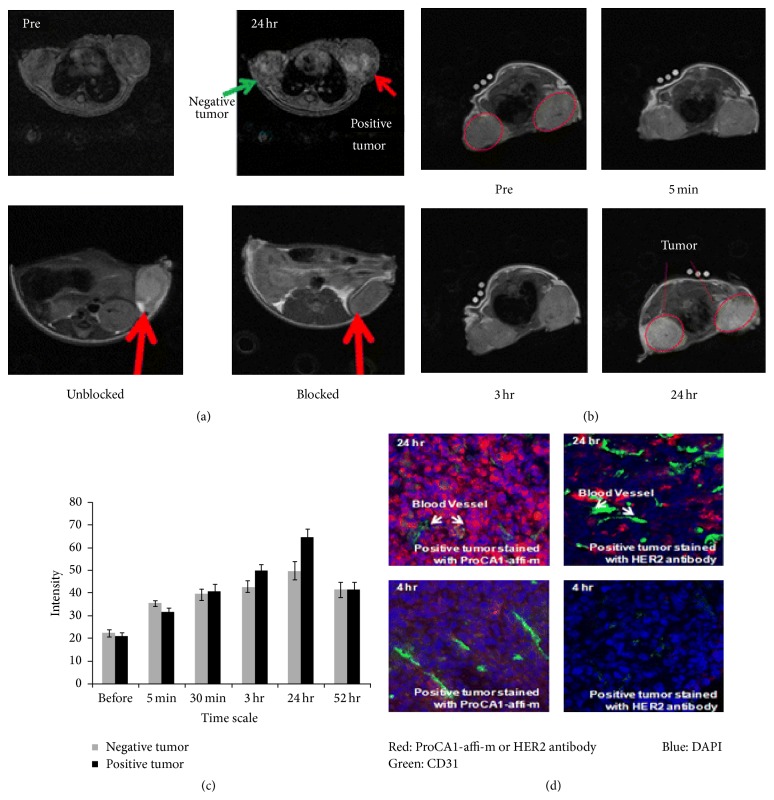
(a) Gradient echo transversal MR images collected prior to injection and at various time points after injection of 3.0 mM ProCA1-affi342-m which is PEGylated ProCA1-affi342 targeting to HER2 in HEPES saline tail vein. The MRI signal on the positive tumor (SKOV-3,* right*) exhibits significant enhancement at 24 h after injection. Blocking results confirm the specific binding of ProCA1-affi342 to HER2-positive tumors. (b) Enhancement changes of MRI intensity in tumors and at 24 h after injection; highest enhancement was observed. (c) MR imaging of contrast agent ProCA1-affi1907 targeting to EGFR specifically. (d) Immunostaining shows that HER2 targeted ProCA1-affi342 has better tumor penetration than HER2 antibodies. Reprinted, with permission, from [38].

In contrast to conventional DCE-MRI, molecular MRI depends on the specificity and amplified action of both contrast agents and targeted molecular probes to improve its sensitivity and can assess molecular events in both normal and tumor cells in vivo. Proteins associated with the physiopathology and biology of breast cancer, such as estrogen, p53, HER2, Ki-67, VEGF, progesterone, and EGFRs, represent specific molecular targets for molecular MRI of breast cancers (Figure 2) [36–38].

### 3.2. PET, SPECT, PET/MRI, and Computed Tomography (CT)

**Figure 3:**
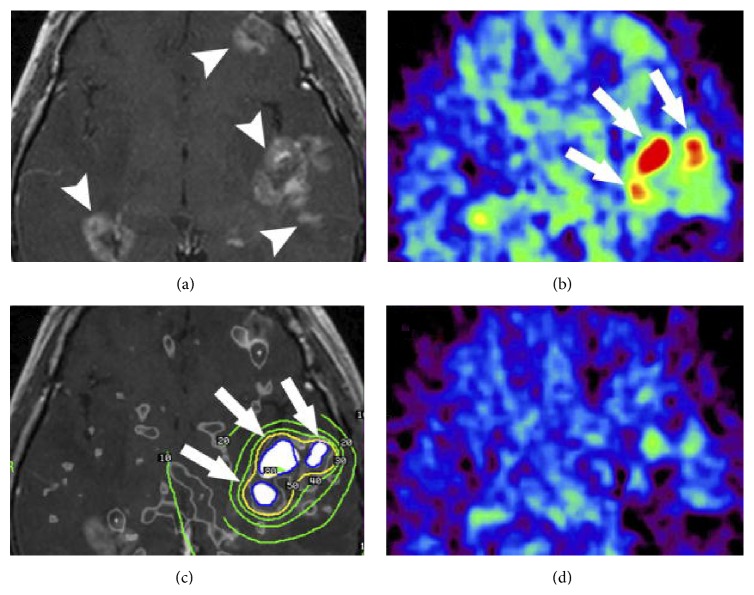
Representative plan for GK surgery treating locally recurred brain metastases with guidance by ^11^C methionine- (MET-) PET/MRI fusion imaging. The patient was a 42-year-old woman with brain metastases from breast cancer who had received 4 previous treatments with GK. (a) A big discrepancy was clearly seen between the area with good contrast enhancement on the T1-weighted MR image (indicated by arrowheads) and (b) the area with elevated MET uptake (indicated by arrows). (c) The PET-MRI fusion image was rendered after constructing a binary image (shown in white), indicating areas with MET uptake 1.4 times higher than that in the contralateral brain. (d) The GK strategy was to administer a higher radiation dose to areas with higher MET uptake. ^11^C methionine uptake was markedly reduced in a MET-PET image obtained 6 months after the GK (i.e., good control of active cancer). Reprinted, with permission, from [40].

For current molecular imaging of tumor receptors, PET and SPECT appear to be more feasible than MRI because they use nuclide tracers with high specific activity [39]. PET requires positron-emitting radionuclides, such as ^18^F, ^11^C, ^13^N, ^94m^Tc, ^120/124^I, ^110^In, ^66/68^Ga, and ^61/64^Cu, while SPECT uses nuclear imaging *γ* emitters, such as ^99m^Tc, ^123/131^I, ^67^Ga, and ^111^In [39]. As a whole imaging modality, ^18^F-fluorodeoxyglucose-PET, a highly investigated and clinically used whole-body imaging modality, provides accurate diagnostic information in patients with breast cancer [25]. Hybrid PET/MRI imaging systems are being developed and promise to improve the diagnostic work-ups of patients with recurrent breast cancer. PET/MRI potentially provides high diagnostic accuracy, soft tissue resolution (via MRI), and specificity (owing to the metabolic information obtained from PET). It may increase diagnostic accuracy after gamma knife (GK) surgery (Figure 3) [25, 40].

**Figure 4:**
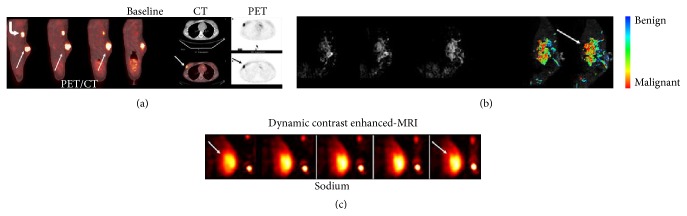
Multimodality PET/CT, dynamic contrast-enhanced (DCE), and sodium MR images on a 35-year-old female with stage III breast cancer before preoperative systemic therapy. (a) There is a marked uptake of ^18^FDG PET within the lesion (SUV) = 6.7. (b) Also rapid uptake of contrast agent within the lesion is shown on the DCE-MR image with a volume (32 mm^3^). (c) There was high total sodium concentration (42 mM) within the lesion. Reprinted, with permission, from [41].

Multimodality imaging, which combines the advantages of different imaging techniques, provides more information on early diagnosis than other systems and allows dynamic monitoring of the responses of the breast cancer to drugs.  Figure 4 presents a typical case in which PET/CT was used. In this case, a 35-year-old woman with an infiltrating breast ductal carcinoma responded to therapy quantified by using a standardized uptake value assessment (SUV) (Figure 4) [41].

### 3.3. Targeted Ultrasound Imaging

Ultrasound imaging discriminates between fluid-filled and solid tissue structures. However, it is not suitable for detailed evaluation of physiopathological changes such as angiogenesis and breast inflammation. A solution to this deficiency is the use of ultrasound contrast agents (UCAs) [42]. Current clinically approved UCAs basically consist of gas microbubbles encapsulated in protein or liposomal carriers [43, 44]. Attempts have been made to produce targeted UCAs. Antibodies, peptides, and other targeting moieties can be bound to microbubbles to target molecules to breast cancer cells that promote angiogenesis and inflammation. One study demonstrated that UCAs targeting HER2, which is dysregulated or overexpressed up to 100-fold in certain subtypes of breast cancer, could generate high-quality ultrasound images of the breast cancer with sufficient mean pixel intensity [42].

### 3.4. Molecular Optical Imaging

Molecular optical imaging offers the possibility of noninvasive, inexpensive, and highly sensitive imaging of breast cancers. Most optical imaging approaches are based on the intrinsic contrast of major tissue chromophores such as hemoglobin, water, and lipids. The main challenges of optical imaging are depth penetration and signal quantification, as well as the development, validation, and approval of relevant optical imaging agents for human use. Light penetration in tissue is limited. However, with the application of near-infrared light, along with the development of more sensitive detection equipment, light penetration in human tissue is now up to 15 centimeters.

Another exciting application of optical technology is the combination of optical imaging with other imaging modalities. Recent efforts have been made to equip hybrid optical imaging with acoustic ultrasound, which may improve the sensitivity and specificity of molecular optical imaging in breast cancer [45].

## 4. Basic Concept of Thermal Ablation and Hyperthermia in Breast Cancer

Thermal ablation enables treatment of localized small breast tumors (2 to 3 cm) [46, 47]. The typical thermal ablation approach is radiofrequency ablation (RFA), which is produced by frictional heating; RF-connected electrode tips placed in the lesion initiate ionic agitation, which generates heat and ultimately destroys cells [48, 49]. RFA was initially used to treat metastatic cancer in the liver and subsequently to treat breast cancer [50]. It is a promising nonsurgical treatment for breast cancer, with reported technical success rates ranging from 76% to 100% [46, 47, 51].

Although there are no standard guidelines for selecting patients for RFA based on tumor size, studies report consistent complete destruction of breast cancers up to 2 cm in diameter; tumors larger than 5 cm usually should be excluded [52, 53]. Patients diagnosed with a single small biopsy-confirmed invasive breast carcinoma are ideal candidates, while extensive diseases including invasive lobular carcinoma and multifocal or multicentric breast lesions are contraindications for curative RFA [54].

Hyperthermia or mild heat at body-tolerable temperatures of 40–44°C has become an effective means of enhancing the efficacy of radiotherapy or chemotherapy in various tumor types [55, 56]. The benefits of hyperthermia combined with radiotherapy or chemotherapy have been documented by several studies of primary and recurrent breast malignancies [57–60]. Hyperthermia can be performed using antennas or applicators that generate heat or photothermal conversion nanoparticles, which are also chemotherapeutic carriers [61, 62].

## 5. Molecular Imaging-Guided Hyperthermia in Breast Cancer

Technologic advances over the last decade have made it possible to cure primary breast malignancies using percutaneous minimally invasive therapeutic approaches [63]. Despite achievements in hyperthermia-based treatments, applying local hyperthermia to deep-seated tumors remains challenging. For example, in pancreatobiliary malignancies, percutaneous RFA or laser ablation has a high risk of damaging adjacent normal structures such as blood vessels, while high-intensity focused ultrasound (HIFU) cannot be used for deep-seated targets owing to inhibition of HIFU energy transfer by adjacent air-containing structures such as the intestine. Application of intrabiliary RF heating by use of antennas or microthermal generators may solve these problems.

Molecular imaging may be useful in this regard. Recent interest in this technique among both diagnostic radiologists and interventionists has led to the establishment of a new concept, namely, “interventional molecular imaging” [28]. This concept aims to fully combine the advantages of two advanced imaging fields, interventional radiology and molecular imaging, with two primary goals. The first goal is local delivery of molecular imaging probes to targets using image-guided interventional techniques, which should prevent renal and hepatic clearance of the probes, as what occurs with systemically administered probes. The second goal is precise image-guided placement of miniature molecular imagers, such as endoluminal optical imagers, in deep-seated targets. This will allow molecular optical imaging not otherwise achieved via percutaneous approaches because of light loss due to tissue scattering and reflection. Molecular imaging can precisely guide probes and assess the responses of their tumor targets to interventional therapies, which is extremely helpful in advanced treatments such as gene therapy and stem cell therapy.

As a step toward molecular imaging-guided hyperthermia in breast cancer, different imaging techniques have been applied. These include HIFU, nanomagnetic particle-based thermotherapy, and real-time thermal mapping (termed MR thermometry) for monitoring breast tumors treated via RFA.

### 5.1. Molecular Imaging-Guided Thermal Ablation and Hyperthermia

#### 5.1.1. MRI-Guided RFA

**Figure 5:**
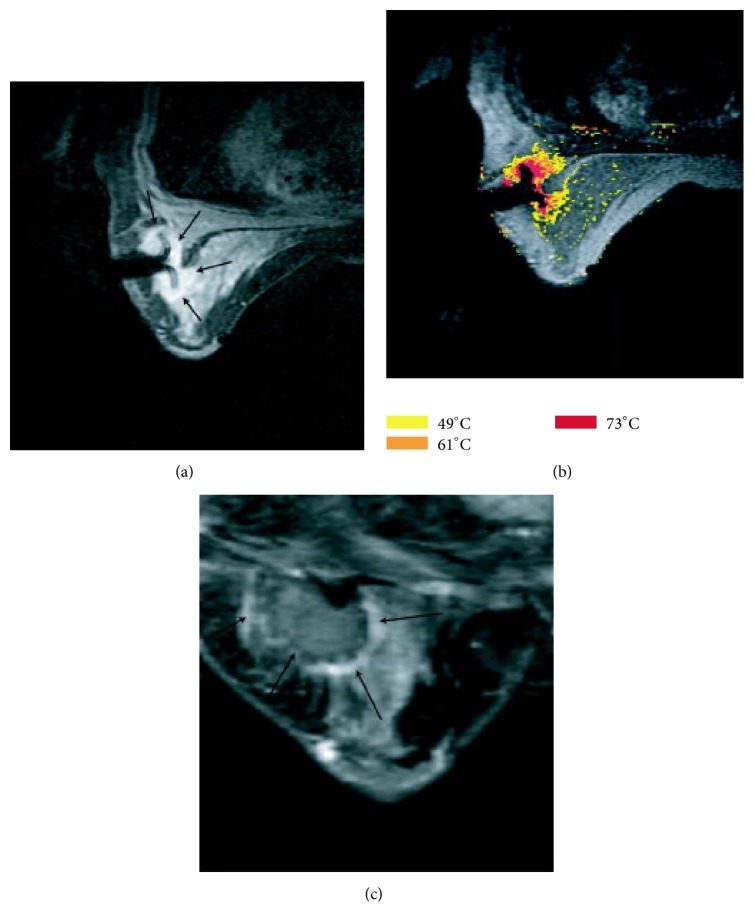
(a) Axial CE three-point Dixon gradient-echo images (150/12.8, 19.8, and 26.8; flip angle = 90°) of the first patient in prone position, showing the enhancing tumor mass (arrow) lateral in the right breast with the hypointense fully deployed LeVeen needle electrode centrally in the center of the mass. (b) Axial CE three-point Dixon gradient-echo images (150/12.8, 19.8, and 26.8; flip angle = 90°) of the same patient in prone position showing the magnetic resonance PRF shift thermomap (yellow zone: 49°C, orange: 61°C, and red 73°C) around the hypointense deployed LeVeen needle electrode centrally in the mass (arrow). (c) Postprocedure CE water-selective, spectral-spatial axial FSE image of the right breast demonstrates a small enhancing rim representing the border of the ablation zone corresponding to fresh scar tissue (arrows). Reprinted, with permission, from [2].

Compared with other imaging techniques, MRI (i) offers more accurate preoperative assessment of the size and extent of the breast cancer, (ii) detects multifocal breast lesions as small as 3 mm [64], (iii) precisely guides the placement of interventional instruments in targeted breast lesions [65, 66], and (iv) allows MR thermometry at controlled ablative temperatures in lesions (Figure 5) [2]. It has been reported that MRI-guided RFA results in complete tumor ablation [46].

#### 5.1.2. MRI Guided Laser-Induced Interstitial Thermotherapy of Breast Cancer

MR-guided, laser-induced interstitial thermotherapy (LITT) can be used to treat primary breast cancer and breast cancer metastases. This technique showed excellent long-term survival results in clinical trials [67, 68] and is a minimally invasive option for treatment of primary breast cancer and especially metastatic tumors.

#### 5.1.3. MRI-Guided HIFU

HIFU is an advanced noninvasive therapeutic technique. Clinical trials consisting of more than 20,000 patients with liver, kidney, or pancreatic cancer, are currently underway in Europe and Asia [69–72]. Because it allows three-dimensional treatment planning and continuous temperature mapping for real-time monitoring of thermal damage in the target zone, MRI-guided HIFU is a valid noninvasive treatment for breast cancer [73]. The current technique requires targets in immobilized organs (e.g., breast and uterus), in which mechanical waves are not attenuated or dispersed by other organs.

Magnetic nanoparticles have been used to assess tumor response and to provide evidence of ablation after treatment. Recent studies support the possibility of generating mild hyperthermia via MRI-guided HIFU for treatment of fibrosarcoma and, in combination with magnetic nanoparticles, treatment of breast cancer [74, 75].

#### 5.1.4. Molecular Image-Guided Radiofrequency-Enhanced Gene Therapy

One of the current limitations of gene therapy is its low capacity to sufficiently transfect/transduce genes/vectors into targets. RF can enhance gene transfer and gene expression by creating local heat. Molecular image-guided RF can also be used to monitor the delivery of nontargeted imaging tracers or therapeutic agents to their specific targets. Yang et al., for example, used intravascular high-spatial resolution MRI to monitor gene delivery to vascular walls [7]. To be visible in MR images, gene vectors are first mixed with an MR contrast agent, and the mixture is locally infused into the vessel wall of the targeted tumor through a balloon catheter. The distribution of contrast agent (and thus of the gene) within the vessel wall is precisely monitored via intravascular high-spatial-resolution MRI [9].

### 5.2. Molecular Imaging in Thermorelated Nanoparticles for Diagnosis and Treatment of Breast Cancers

Nanoparticles are an interdisciplinary topic of interest for research pertaining to the diagnosis and treatment of breast cancer. Nanomaterials can be engineered to serve as targeted molecular imaging contrast agents and antitumor drug delivery vehicles [76]. Specific targeting of nanoparticles allows controlled drug release and enhances drug permeability and retention despite the leaky vasculature of cancerous tissues. The identification of specific molecular and biological pathways in breast cancer has spurred the development of novel nanoscale approaches for diagnosis and treatment of breast cancer [77].

Coencapsulating chemotherapeutic drugs and MRI-visible agents in one carrier provide a valid tool for image-guided, temperature-induced drug release. Hyperthermia-induced release of capsule contents can be monitored indirectly by corelease of an MRI contrast agent once the nanoparticles arrive at the target. Within the nanoparticles, some MRI contrast agents cannot be visualized because of limited water exchange across the lipid bilayer of the cell membrane. However, when the temperature of the phase transition of the cell membrane rises, water exchange increases and/or contrast agent is released from the nanoparticles, which thereby shortens the ^1^H MR relaxation time of the surrounding tissues for generating MR signals.

Magnetic nanoparticles provide a good signal in soft tissue and thus morphological/anatomical information, while MR thermometry evaluates alterations in hyperthermia at different dimensions within the heated tissues [78, 79]. Some investigators coencapsulated doxorubicin and manganese (a T1 MRI contrast agent) in one nanocarrier for molecular MRI-guided drug delivery and, by doing so, created the concept of “dose painting,” which was demonstrated via MRI [80, 81]. A milestone in molecular imaging-guided hyperthermia was the conjugation of magnetic nanoparticles to HER2 monoclonal antibodies to allow specific binding of the nanoparticles to HER2, which is present in approximately 17% of breast cancers. HER2-specific magnetic nanoparticles can carry and release coloaded chemotherapeutic drugs both in vitro and in vivo [26] and have been successfully used to inhibit the proliferation of breast cancer cells [82–84] and in primary systemic therapy prior to surgical intervention [85]. Magnetic hyperthermia and magnetic nanoparticles in alternating magnetic fields could partially or completely destroy small occult lesions, which would limit the extent of or need for surgical intervention [85, 86].

PET and SPECT imaging are often used to visualize nanoparticles delivered to certain types of tumors. Nanoparticles can also be labeled with radionuclides such as ^99m^Tc, ^111^In, ^201^TI, ^18^F, ^123^I, ^131^I, and ^6^Ga [87–90]. By the use of these radioactive nanoparticles, molecular imaging-guided interventions have become feasible.

## 6. Conclusions

Molecular imaging is an excellent technique for early detection and guided targeted therapy of breast cancer. Molecular imaging-guided intervention in combination with hyperthermia can further improve the treatment of this common, worldwide life-threatening malignancy in women. The combination of advanced multidisciplinary technologies such as molecular imaging-integrated hyperthermia and interventional radiology, as well as gene therapy and chemotherapy, provides new options for effective management of breast cancer.

## Figures and Tables

**Table 1:** Highlights of the genomic, clinical, and proteomic features of the four main subtypes of breast cancer.

Subtype	IHC markers [21]	DNA mutations [21]	mRNA expression [21]	Benefit from chemotherapy [22]	Outcome [22]
Luminal A	ER^+^/HER2^−^: 87%; HER2^+^: 7%; TNBCs: 2%; and Ki67: low	PIK3CA (49%); TP53 (12%); GATA3 (14%); and MAP3K1 (14%)	High ER cluster; low proliferation	Low (0–5% pCR)	Good

Luminal B	ER^+^/HER2^−^: 82%; HER2^+^: 15%; TNBCs: 1%; and Ki67: high	PIK3CA (32%); TP53 (32%); and MAP3K1 (14%)	Lower ER cluster; high proliferation	Intermediate (10–20% pCR)	Intermediate or poor

Basal-like	ER^+^/HER2^−^: 10%; HER2^+^: 2%; TNBCs: 80%; and Ki67: high	PIK3CA (7%); TP53 (84%)	Basal signature; high proliferation	High (≥40% pCR)	Poor

HER2E	ER^+^/HER2^−^: 20%; HER2^+^: 68%; TNBCs: 9%; and Ki67: high	PIK3CA (42%); TP53 (75%); and PIK3R1 (8%)	HER2 amplicon signature; high proliferation	Intermediate (25–40% pCR)	Poor

IHC: immunohistochemistry; ER: estrogen receptor; HER2: human epidermal growth factor receptor 2; PR: progesterone receptor; +: positive; −: negative; TNBCs: triple-negative breast cancers; pCR: pathological complete response after neoadjuvant chemotherapy.
